# Capillary force-induced superlattice variation atop a nanometer-wide graphene flake and its moiré origin studied by STM

**DOI:** 10.3762/bjnano.10.80

**Published:** 2019-04-01

**Authors:** Loji K Thomas, Michael Reichling

**Affiliations:** 1 Fachbereich Physik, Universität Osnabrück, Barbarastr. 7, 49076 Osnabrück, Germany; 2 Department of Physics, S. B College, Mahatma Gandhi University, Kerala, 686101, India

**Keywords:** capillary force, graphene, graphite, HOPG, moiré, solid–liquid interface, STM, superlattice

## Abstract

We present strong experimental evidence for the moiré origin of superlattices on graphite by imaging a live transition from one superlattice to another with concurrent and direct measurement of the orientation angle before and after rotation using scanning tunneling microscopy (STM). This has been possible due to a fortuitous observation of a superlattice on a nanometer-sized graphene flake wherein we have induced a further rotation of the flake utilizing the capillary forces at play at a solid–liquid interface using STM tip motion. We propose a more “realistic” tip–surface meniscus relevant to STM at solid–liquid interfaces and show that the capillary force is sufficient to account for the total expenditure of energy involved in the process.

## Introduction

Graphite is a layered material with graphene sheets arranged in ABAB stacking. HOPG is an ordered form of pyrolytic graphite with a high degree of crystallographic orientation of the *c*-axis. Polycrystalline HOPG consists of micrometer-sized grains and has been widely used as a substrate in STM studies [1–3] due to its high conductivity, atomic flatness and chemical inertness [4]. The surface also contains various defects such as cleavage steps, graphite strands, wrinkles/ridges, fiber-like entities, folded-over flakes, broken graphite pieces and other carbon aggregates [5–10]. Graphene, a monolayer of graphite, is the thinnest and strongest material ever known [11–13] and holds immense potential for applications [14–15]. Many applications using graphene requires its electronic structure to be modified at the nanoscale.

Positioning the top layer of a layered material at different orientations about the *c*-axis could produce different electronic surface profiles for the material [16]. Since an STM image is a map of the local electronic density of states (LDOS), such electronic modifications may be visualized in real space. When STM is operated at solid–liquid interfaces, the capillary force due to the meniscus formed between the tip and the surface could be utilized for mechanically manipulating graphene flakes on the surface [17]. According to calculations, the pressure within the meniscus could be large enough to cause small deformations in crystalline materials such as graphite [17–18]. Here we try to use the capillary force to displace a nanometer-wide graphene flake with the intent of altering the superlattice on the flake. The change in the superlattice periodicity is then used to validate the moiré origin of the superlattices. We also describe the theory behind the plausible cause of the rotation of the flake based on a more “realistic” meniscus pertinent to the situation of a solid-liquid STM measurement.

## Results and Discussion

Apart from various defects [5–9], hexagonal superlattices are the most frequently observed planar artefacts found on HOPG(0001) during STM imaging [19–22]. It was proved by Xhie et al., based on a direct measurement of the grain orientations at a grain boundary, that a superlattice is a result of mechanical rotation of the top layer; however, no live change in the periodicity was reported [19]. The periodicity *D* of the resulting moiré pattern is given by

[1] D=d/(2sin(θ/2)).

Here θ represents the rotational angle between the lattices and *d* is the lattice constant, which is 0.246 nm for the graphite lattice [23]. Once θ is found, the orientation Ф of the superlattice relative to that of underlying atomic lattice can be found using

[2] Φ=30°−θ/2 .

The observed superlattice periodicities, corrugation amplitudes and the dependence on bias voltages have been summarized in a review elsewhere [24]. There have been reports both in support [21,25] as well as against [26–27] the simple moiré theory. Changes of the superlattice periodicity in space [25] and time [17] have been reported before. However, there has been no report on a real-time observation of a change in the periodicity with concurrent and direct measurement of the orientation of the top graphene layer before and after its rotation. In our report, this was made possible only due to a lucky observation of a small nanometer-wide graphene flake. Here we show how the capillary forces acting at the tip–surface meniscus [28] could be utilized to achieve a rotation of a nanometer-wide graphene flake and induce a change in its superlattice periodicity in real-time. The perfect agreement with calculations of the periodicities before and after rotation provides a direct experimental verification of the moiré theory. Note that the capillary force could induce a translation or other type of deformation of the flake and we have no real control over this aspect; however, here the flake was found to be rotated.

**Figure 1:**
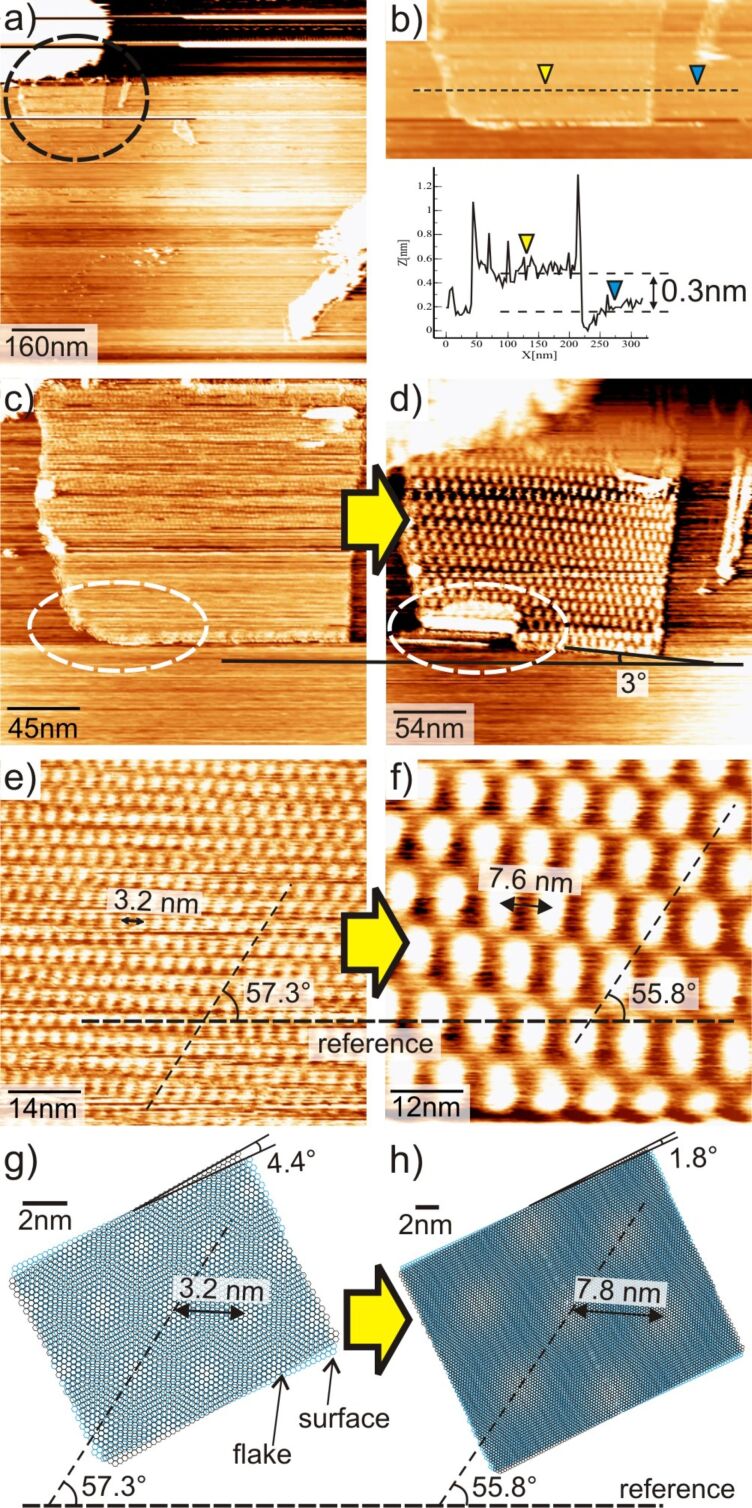
(a) STM image of a 160 nm wide, one-layer thick graphene flake (encircled); (b) cut out portion and height profile along the dashed line on the graphene flake. Zoomed-in region imaged by STM: (c) before rotation of the flake, (d) after a rotation by ca. 3°. (e) and (f) are STM images showing the lattices within the flake in (c), and (d) respectively. (g) and (h) are models for the superlattices in (e) and (f) respectively. Imaging parameters for (a,c,e) are 190 mV, 0.6 nA and (d,f) are 80 mV and 0.6 nA.

Superlattices are no rarity, and in fact, this is a common occurrence during STM imaging of bare or solution-covered HOPG surfaces. It has been reported that moiré patterns could be easily produced, albeit with no control over their periodicity, by immersing HOPG in organic solvents such as dichloroethane; whereas dry-prepared HOPG samples seldom displayed moiré patterns [29]. Roy et al. demonstrated the STM manipulation of folding of graphene under UHV conditions [30], although tip-driven surface layer deformation may occur more easily in air than in vacuum [31]. Under humid or liquid conditions, capillary forces are present and might offer a substantial amount of force for rotation or translation of the top layer [17,31–33]. The presence of an organic solution, thus, may facilitate the rotation of the top-layer graphene yielding superlattices on graphite [7,17,29]. Here, we report a rare occurrence of an isolated, nanometer-wide graphene flake and its live transformation from one superlattice to another. We noticed the superlattice on a graphene flake at the interface of a dilute organic solution (3,4,5-tris(octyloxy)benzamide in 1,2,4-trichlorobenzene (C_6_H_3_Cl_3_)) and graphite. Figure 1a shows an STM image of a graphite superlattice atop a single-layer graphene flake (circled) on a graphite(0001) surface. The graphite flake is only about 160 nm wide with an apparent height of ca. 0.3 nm as shown in the height profile in Figure 1b, which is close to the interlayer spacing of graphite (0.34 nm) [23]. The superlattice is limited to the region of the graphene flake as seen in Figure 1a. Figure 1c is a zoomed-in region on the graphene flake imaged by STM, exhibiting a hexagonal superlattice of periodicity 3.2 nm that is aligned at an angle of 57.3° with the horizontal as illustrated in Figure 1e. Here, all angles are measured with respect to the horizontal as a reference line. At this point, we zoomed out and scanned the area, but without imaging, at a slightly higher current of 1 nA (i.e., at a closer tip–surface distance) for less than one up-scan, and thereafter zoomed-in back to the same area, to see whether the capillary forces at the interface could perturb the graphene flake. The STM image taken after this process is shown in Figure 1d (and cut-out part in Figure 1f) where the superlattice periodicity has been changed to 7.6 nm with a concurrent change in the angle to 55.8°.

It seems that during the above procedure, the capillary force might have caused a small clockwise rotation of the graphene flake by about 3° [28,30] with respect to the reference line (Figure 1d). The impact also caused some damage on the lower left region of the flake (dashed white ellipse) in the form of tearing of the graphene sheet along a length of *b* = 15 nm (see also Figure 3c). Now, if we assume that the superlattices are caused by moiré rotation and Equation 1 and Equation 2 hold, then for the initial superlattice of *D*_1_ = 3.2 nm, the rotational angle θ_1_ between the graphene flake and the bulk layer beneath, can be found using Equation 1, which gives a value of 4.4° (Figure 1g). Similarly, after the transition to the larger superlattice with *D*_2_ = 7.6 nm, the corresponding angle can be found which is θ_2_ = 1.8° (Figure 1h). That is, calculation suggests that the angle of the top graphene flake with respect to the bulk surface has changed from an initial position at 4.4° to the final position at 1.8°, i.e., Δθ = 2.6°. This is close to the direct measurement on the STM image (Figure 1d) that appears to be a clockwise rotation of 3°. We make a lattice model that is shown in Figure 1g for the first superlattice of Figure 1e. The black lattice represents the atomic lattice of the graphene flake and the blue one that of the underlying bulk graphite surface. Now, it can be seen that, in the model, a clockwise rotation of Δθ = 2.6° of the black lattice results in a superlattice with a periodicity of 7.8 nm as illustrated in Figure 1h. Comparing with the experimental result in the STM measurement of Figure 1f, the periodicity is found to be 7.6 nm. Thus, there is a perfect agreement between the periodicities in the model and the STM images of Figure 1e and Figure 1f. The small discrepancy between STM measurements and the calculation could be attributed to thermal drift in STM imaging under ambient conditions.

A further verification of the moiré assumption is possible by direct measurement of the orientation of the flake with a reference direction before and after rotation. If the assumption of the moiré rotation is right, one should, in addition to the agreement in the periodicities in the STM images, should also agree on the orientation of the respective superlattices with respect to a reference line. This again is easily proved comparing the STM image in Figure 1f and the model in Figure 1h where the superlattice in both cases is oriented 55.8° relative to the reference line. Thus there is a perfect match between the calculated and experimental values for both the periodicity as well as the angular dependence. From the STM images of Figure 1c and Figure 1d, it can be directly verified that a clockwise rotation of Δθ = 3° (2.6° from calculation) has occurred.

**Figure 2:**
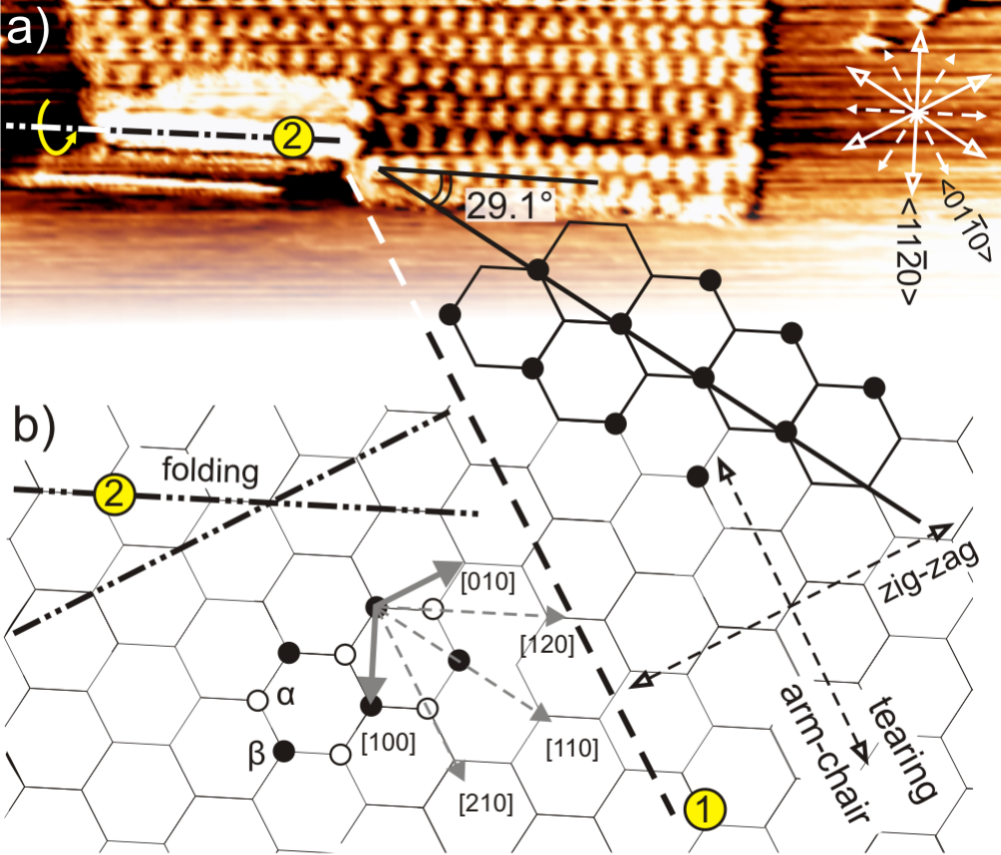
(a) Cut-out portion of the STM image showing the final superlattice with [210] tearing direction numbered as (1) and the [120] folding axis numbered as (2). (b) graphite lattice of the top layer with tearing direction and folding axis marked in accordance with (a). Short grey arrows represent the graphite unit vectors. The graphite lattice is not drawn to scale to that of the STM image.

With the rotation of the graphene sheet, there is also a tearing on the flake visible on the lower left region (Figure 2a). It thus seems that the impact of the capillary force was highest on the lower left part of the flake. The torn part appears also to be partially folded over, leaving an empty region of about 72 × 9 nm^2^ area where no trace of the superlattice could be found. It has been reported previously that energetically preferred directions exist for folding and tearing of graphene layers [5,9,21,34]. This is depicted in Figure 2b. The designation into α- and β-carbon atoms is peculiar to STM imaging of the graphite(0001) surface where only the β-sites are visible to STM [35–37]. Since from Figure 1h, the angle between the two top-most graphene atomic lattices of the final superlattice is known (1.8°), the tearing and folding directions can now be verified using Equation 2. The equation predicts an angle of 29.1° between the superlattice and the top atomic lattice. Based on this, a model of the top graphene atomic lattice is superimposed on the STM image in order to identify the tearing and folding directions of the graphene flake. According to this construct, the lattice gives the tearing direction (numbered (1)) and folding axis (numbered (2)) along two arm-chair crystallographic directions that differ by 120°, in agreement with previous studies [5,9,21,34].

## Theory

There are three significant forces existing at a solid–liquid interface [17]: (a) van der Waals; (b) electrostatic interaction due to the applied potential difference between the tip and sample [38–43] (or tip and graphene [28]), and (c) capillary forces due to the Laplace pressure generated by the formation of a highly curved fluid meniscus between the tip and the surface [29,44–45]. The capillary force is known to be many orders of magnitude higher than electrostatic or van der Waals interactions [17].

**Figure 3:**
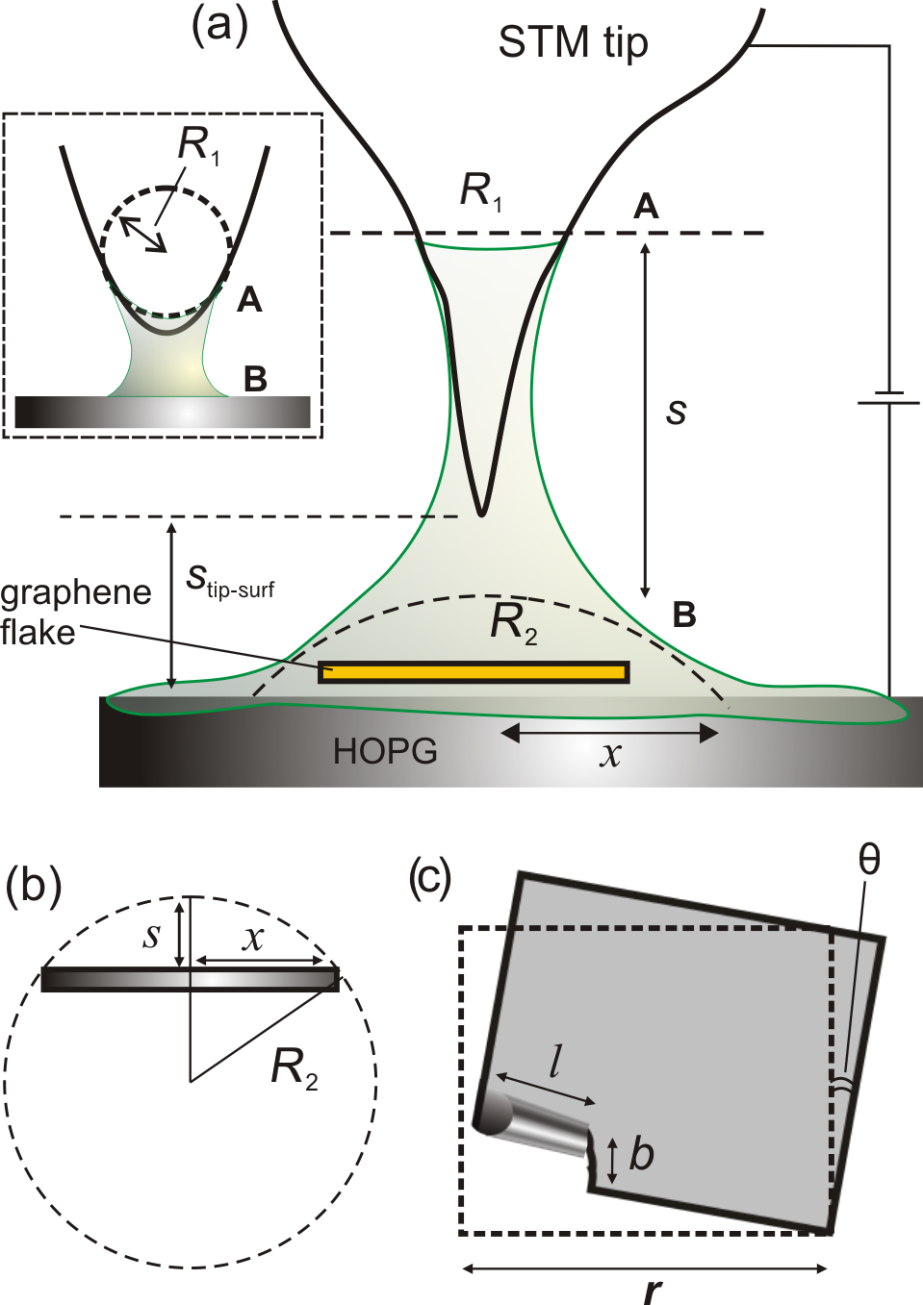
(inset) Sphere–plane geometry with an atomically sized sphere of radius *R*_1_ at the tip side (A-side), (a) proposed model suitable for STM solid–liquid interface with the meniscus possessing a radius of curvature *R*_2_ at the surface (B-side) where *R*_2_ << *R*_1_, (b) sketch for calculating the radius *R*_2_ in terms of the spread (*x*) of the liquid on the surface, (c) schematic of the graphene flake that has undergone rotation, tearing and partial folding.

The graphene flake of area 160 × 182 nm^2^ has undergone three processes as shown in Figure 3c, i.e., a rotation, a tearing along the arm-chair direction for an extent *b* = 15 nm, and a partial folding of a small region of axial length *l* = 72 nm. The expenditure of energy for tearing, folding and rotation of the flake can be calculated from the known values of bond energies (see Table 1). The C–C bond energy is 4.9 eV [4,17,44–45] from which the energy required to break the bonds along the arm-chair direction can be found, which amounts to 172 eV. The interlayer binding energy in graphite is 44 meV/atom which is the van der Waals barrier that needs to be overcome for rotation to happen [45]. Theoretical studies showed that the energy barrier for rotation of graphene flakes on graphite is of the order of *k*_B_*T* [46–48], and for small angles, 25 meV/atom [47] will be a good approximation. Therefore the rotational barrier for the entire area of the flake is 28 keV. This energy is in kiloelectronvolts due to the very large number of atoms involved (see Table 1). The folding energy is calculated in analogy to the energy of a collapsed carbon nanotube [17,30,49], *E*_fold_ = *k*·*a*·*l*/2*r*^2^ where *k* is the curvature modulus (*k* = 1.4 eV for CNTs with radii smaller than 2.4 Å), *a* the arc length which is ≈ *b* = 15 nm, *l* the length of the curved region of 72 nm, and *r* the radius of curvature ca. 2.5 nm (*a* = 15 nm and *r* = 15/2π), respectively. This equation can also be written as *E*_fold_ = *k2*π*r*·*l*/2*r*^2^ = π*k*·*l*/*r*, which yields around 126 eV for the folding energy barrier. Therefore, the total energy spent in rotation, tearing and folding of the flake adds up to about 28.3 keV.

**Table 1:** Expenditure of energy in various processes.

bond	energy	process	number of bonds / atoms / dimension	total energy

*E*_rotat_/atom	<25 meV/atom	rotation	area of flake = 160 × 182 nm^2^ ≡ 112 ×10^4^ atoms	28 keV
*E*_C–C_	4.9 eV/bond	tearing	*b* = 15 nm ≡ 35 bonds (along arm-chair)	172 eV
*E*_fold_		folding	*b* = 15 nm, *l* = 72 nm	126 eV

The tip–surface meniscus is similar to the pointed end of a nail placed on a thin layer of water very close to the surface but without touching it. So, when the nail is moved horizontally over the surface, the meniscus at the nail side is also dragged along due to the capillary force holding the meniscus together. This way the meniscus can exert a force on a flake on the surface when the nail moves over the surface. In order to explain the capillary forces at a solid–liquid interface pertinent to the STM scenario, we propose a modified model as shown in Figure 3a. In contrast to previous studies in STM [17] and AFM [50] or about problems in capillary mechanics, we point out a major difference in the meniscus formation at an STM tip–surface interface. Here, the distance *s*, between the surface and the STM tip is not decided by the requirement for meniscus formation, rather it is defined by the STM operating parameter namely the set point current (in constant-current mode), which fixes the tip–surface separation *s*_tip–surf_. The STM tip is usually immersed in the liquid and therefore the distance *s* is always greater than or equal to *s*_tip–surf_. Further, the meniscus is not static but in motion due to the raster scanning of STM tip.

The capillary force between two objects of spherical and planar geometry can be approximated as *F* = 4πγ*R* where *R* is the radius of the sphere (not the meniscus), and γ is the surface tension of the liquid [32,50], which is 39.1 mN/m for 1,2,4-trichlorobenzene. This is the maximum force called the adhesive force occurring for small values of *s*, where *s* is the separation between the plane and the sphere. In the case of a sphere–sphere geometry, with radii of curvatures of the spherical bodies on either sides being *R*_1_ and *R*_2_, the radius *R* can be replaced by an effective radius R* = *R*_1_*R*_2_/(*R*_1_ + *R*_2_) making it an equivalent case of a plane interacting with a sphere having an effective radius R*, thus the equation reads *F* = 4πγR* [32].

The application of capillary equation to STM scenario usually presumes a meniscus with the tip side (A) possessing a spherical geometry and the surface part (B) to be flat, constituting a sphere–plane geometry [17] (see inset of Figure 3). Since the tip edge is expected to possess a “single-atom” or few-atom termination for obtaining high-resolution images, the radius of the sphere should be in the range of a few angstroms. This is in contrast to meniscus formation in sphere–plane capillary systems where the size of the sphere is appreciable, i.e., in the range of micrometers [32]. The STM tip is actually immersed in the liquid the diameter of which is around 250 μm (Figure 3a). Therefore, the model illustrated in the inset of Figure 3 is not suitable for STM operation at a solid–liquid interface. In STM experiments, the meniscus is macroscopically visible with dimensions equal to the diameter of the tip (A-side). Typical Pt/Ir tips used are 0.25 mm in diameter. The lateral spread of the liquid on the surface side (B-side) extents much beyond than that on A-side. In order to take into account the larger volume of the meniscus at the surface side, we propose a plane–sphere geometry with the sphere at the B-side and plane at the A-side since the curvature is higher on the surface side (A-side). Mathematically, this is the equivalent case of a plane interacting with a sphere with an effective radius R*. It can be imagined that the thin film of liquid on the surface acts like an object that supports the meniscus with a curvature, *R*_2_. That is the spread (*x*) that contributes is only the volume of the liquid forming a curved meniscus, and not the regions where the liquid meniscus is flat (see Figure 3a). Now, as for the case of a plane–sphere geometry [32], the curvature of the meniscus *R*_2_ can be taken as the curvature of the sphere. From the geometry of the tip–surface interface as shown in Figure 3a, *R*_1_ >> *R*_2_ and the effective radius R* = *R*_2_. With this, the equation becomes *F* = 4πγ*R*_2_. The physical meaning of this is that the surface with higher curvature, i.e., smaller radius of curvature (here *R*_2_) predominantly contributes to the capillary force arising from the meniscus.

**Figure 4:**
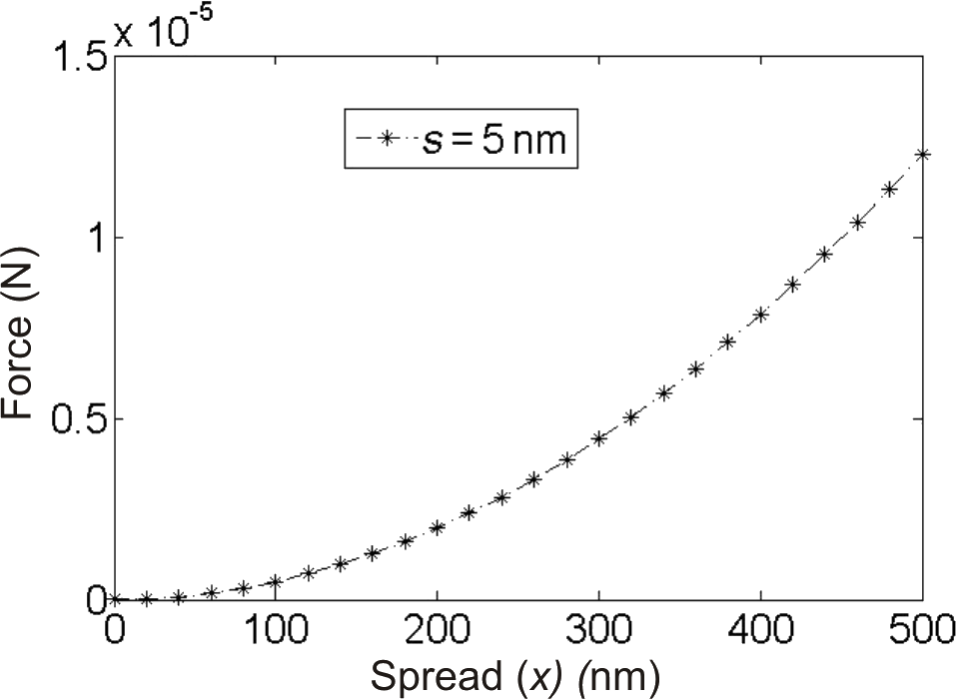
Force-vs-distance (*x*) curve according to Equation 3 plotted for *s* = 5 nm.

Unlike the tip radius *R*_1_, the radius *R*_2_ or the extension (*x*) of the meniscus on the surface (B) is not known. So, we resort to a range of values for *R*_2_ by varying the extension (*x*) of the meniscus on the surface as illustrated in Figure 3b, and plot the force-vs-distance (*x*) curve that is shown in Figure 4. For this we modified the force equation using *R*_2_ = (*s*^2^ + *x*^2^)/2*s* based on the construct shown in Figure 3b. With this substitution, the equation for the capillary force becomes

[3] $$F\;=\;4\pi \gamma \left(s^{2}+\;x^{2}\right)/2s.$$

The curve in Figure 4 is plotted for *s* = 5 nm, although, in principle, it could be any distance that supports a meniscus in the STM context with *s* ≥ *s*_tip–surf_. The energy associated with the capillary force is equal to the work done 
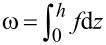
 [51] when the surface tension lifts a liquid upward in a tube over a distance *h*. Here, unlike the rise of liquid in a capillary tube, most of the energy is utilized for rotation of the flake. So, we estimate the force required for the rotation and hence the spread *x* or *R*_2_ required to support it. Since the energy expenditure for rotation is about 28 keV, using τθ = *rF*θ, where τ is the torque, *r* = 1820 Å (see Table 1), we can calculate the force required to rotate the graphene flake by an angle θ = 2.6°, which is about 0.05 × 10^−5^ N. In the force-vs-distance plot, this force can be traced to very small values of the spread (*x*) on the surface side. Note that the spread we refer to is only the volume of the liquid that has a high curvature. Thus, capillary force arising from a meniscus with even a very small amount of spread could provide adequate force for the rotation of the flake.

## Conclusion

A modification of the superlattice on a nanometer-wide graphene flake has been achieved by STM manipulation using the capillary forces at play at a graphite–liquid interface. The calculated periodicities and orientations of the initial and final lattices are in perfect agreement with values extracted from STM images, validating the moiré theory. A new “realistic” model for the capillary force at the interface pertinent to STM at solid–liquid interfaces is introduced. We showed that the capillary force alone can account for the entire expenditure of energy.

## Experimental

STM imaging: A freshly cleaved sample of highly oriented pyrolytic graphite (HOPG, ZYB grade, SPI supplies, West Chester, PA, USA) was used. STM images were taken in the constant-current mode under ambient conditions with a compact STM (easyScan, Nanosurf AG, Liestal, Switzerland). Mechanically sharpened Pt/Ir 80/20% wires (Goodfellow Cambridge limited, Huntingdon, United Kingdom) were used as STM tips. The solution was drop-cast on HOPG, and the images were taken at a solution–graphite interface with a thin meniscus between the tip and the sample. By imaging the atomic structure of the bare graphite, the scanner was calibrated in regular time intervals so that the precision of measurements are solely limited by thermal drift. The ambient temperature is stabilized to be within ±1.0 °C of room temperature and the scanner is always given time to thermally equilibrate and mechanically relax to reduce thermal drift and piezo creep to a minimum during measurements. Typical tunnelling conditions were *V*_b_ = 0.05 to 0.19 V and *I*_t_ = 0.6 nA. Images represent raw data and were analyzed using the WSxM software [52].

## Supporting Information

File 1 STM image of graphite(0001) surface showing β atoms, STM image of moiré pattern at arachidic acid-HOPG interface.
