# Supplementary material for: Capillary force-induced superlattice variation atop a nanometer-wide graphene flake and its moiré origin studied by STM
Source: Beilstein J Nanotechnol. 2019 Apr 1;10:804–10. doi: 10.3762/bjnano.10.80 (PMC6466726; doi:10.3762/bjnano.10.80)
Supplement: File 1 — STM image of graphite(0001) surface showing β atoms, STM image of moiré pattern at arachidic acid-HOPG interface. [file Beilstein_J_Nanotechnol-10-804-s001.pdf]

BEILSTEIN JOURNAL OF NANOTECHNOLOGY

Supporting Information

for

Capillary force-induced superlattice variation atop a nanometer-wide graphene flake and its moiré origin studied by STM

Loji K. Thomas and Michael Reichling

Beilstein J. Nanotechnol. **2019,** *10,* 804–810. doi:10.3762/bjnano.10.80

STM image of graphite(0001) surface showing β atoms, STM image of moiré pattern at arachidic acid-HOPG interface

License and Terms: This is a supporting information file under the terms of the Creative Commons Attribution License (http://creativecommons.org/licenses/by/4.0). Please note that the reuse, redistribution and reproduction in particular requires that the authors and source are credited.
The license is subject to the *Beilstein Journal of Nanotechnology* terms and conditions: (https://www.beilstein-journals.org/bjnano)

The following two images introduce two simple aspects in STM imaging of HOPG:
(Figure S1) STM image only every other carbon atom (β atoms), (Figure S2) Rotation of top layer is visible as a moiré pattern.

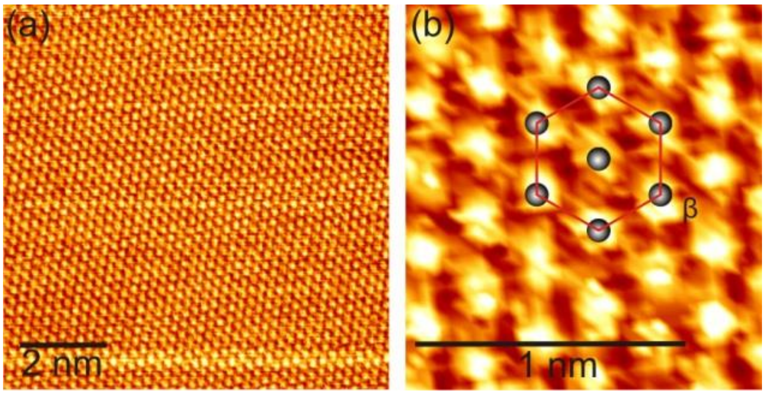

Figure S1: (a) STM image of a graphite(0001) surface, (b) STM images only every second carbon atom (called β) of the (0001) surface.

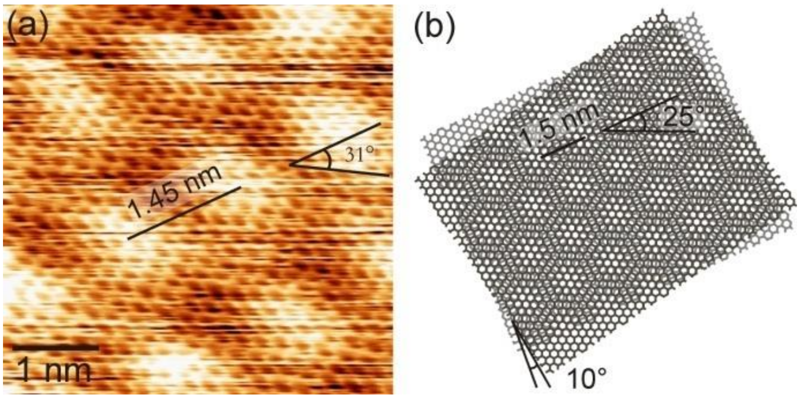

Figure S2: Illustration of graphite superlattice formation by moiré rotation: (a) STM image showing both the superlattice and underlying graphite basal plane obtained at arachidic acid-HOPG interface (imaging parameters 0.34 V, 0.87 nA). The shorter lattice (0.246 nm) represents graphite surface and the larger bright blobs represent the superlattice (1.45 nm), (b) the observed periodicity can be explained, apart from small thermal drift in the image, by rotation of the topmost graphene layer.
